# *Streptococcus suis* serotype 2 strains isolated in Argentina (South America) are different from those recovered in North America and present a higher risk for humans

**DOI:** 10.1099/jmmcr.0.005066

**Published:** 2016-10-31

**Authors:** Raquel Callejo, Han Zheng, Pengcheng Du, Monica Prieto, Jianguo Xu, Gustavo Zielinski, Jean-Philippe Auger, Marcelo Gottschalk

**Affiliations:** ^1^​ Instituto Nacional de Enfermedades Infecciosas, Buenos Aires, Argentina; ^2^​ Collaborative Innovation Center for Diagnosis and Treatment of Infectious Diseases, State Key Laboratory for Infectious Disease Prevention and Control, National Institute for Communicable Disease Control and Prevention, Chinese Center for Disease Control, Changping, Beijing, PR China; ^3^​ Institute of Infectious Diseases, Beijing Ditan Hospital, Capital Medical University, Beijing Key Laboratory of Emerging Infectious Diseases, Beijing, PR China; ^4^​ Instituto Nacional de Tecnologia Agropecuaria (INTA), Marcos Juárez, Córdoba, Argentina; ^5^​ Swine and Poultry Infectious Disease Center, (CRIPA), Faculty of Veterinary Medicine, University of Montreal, St-Hyacinthe, Quebec, Canada

**Keywords:** Streptococcus suis serotype 2, swine, zoonosis, virulence factors, multilocus sequence typing, sequencing

## Abstract

**Introduction:**

*Streptococcus suis* serotype 2 is an important swine pathogen and emerging zoonotic agent causing meningitis and septicemia/septic shock. Strains are usually virulent (Eurasia) or of intermediate/low virulence (North America). Very few data regarding human and swine isolates from South America are available.

**Case presentation:**

Seventeen new human *S. suis* cases in Argentina (16 serotype 2 strains and a serotype 5 strain) are reported. Alongside, 14 isolates from pigs are analyzed: 12 from systemic disease, one from lungs and one from tonsils of a healthy animal. All human serotype 2 strains and most swine isolates are sequence type (ST) 1, as determined by multilocus sequence typing and present a *mrp^+^/epf^+^/sly*^+^ genotype typical of virulent Eurasian ST1 strains. The remaining two strains (recovered from swine lungs and tonsils) are ST28 and possess a *mrp^+^/epf*^−^*/sly^−^* genotype typical of low virulence North American strains. Representative human ST1 strains as well as one swine ST28 strain were analyzed by whole-genome sequencing and compared with genomes from GenBank. ST1 strains clustered together with three strains from Vietnam and this cluster is close to another one composed of 11 strains from the United Kingdom.

**Conclusion:**

Close contact with pigs/pork products, a good surveillance system, and the presence of potentially virulent Eurasian-like serotype 2 strains in Argentina may be an important factor contributing to the higher number of human cases observed. In fact, Argentina is now fifth among Western countries regarding the number of reported human cases after the Netherlands, France, the UK and Poland.

## Introduction

*Streptococcus suis* is an important swine pathogen mainly causing septicemia, meningitis and arthritis (Gottschalk, 2012). In addition, it is an emerging zoonotic agent responsible for septicemia with or without septic shock and meningitis (Gottschalk, 2012). During the last decade, the number of human cases due to *S. suis* has increased, and while most sporadic human cases of infection occur following close occupational contact with pigs/pork products, particularly in Western countries, important outbreaks have been recorded in Asia. In the latter, the general population is at risk due to consumption of raw pork products as part of traditional dishes. Indeed, *S. suis* infections are considered to be among the most frequent causes of adult meningitis in Asia (Wertheim *et al.*, 2009).

Although different serotypes have been described, serotype 2 is the most commonly isolated from diseased pigs; it also represents more than 95 % of human cases worldwide (Goyette-Desjardins *et al.*, 2014). However, important differences in virulence of isolates within this serotype have been described (Auger *et al.*, 2016). It has been shown that Eurasian strains generally present a higher virulence potential than those from Canada and the USA, two countries that together are the second most important swine producers worldwide after China (Gottschalk, 2012).

Multilocus sequence typing (MLST) has been used worldwide to determine the sequence types (STs) of *S. suis* strains, thus allowing the gathering of further information regarding their genetic diversity and evolution (Goyette-Desjardins *et al.*, 2014). More recently, studies have begun combining data obtained from MLST with the presence or absence of different *S. suis* serotype 2 virulence-associated markers such as muramidase released protein (*mrp*), extracellular protein factor (*epf*), and suilysin (*sly*), to compare ST data with pathotypes. ST1 strains, which are usually more virulent and possess a genotypic *mrp^+^/epf^+^/sly^+^*pathotype profile, are mostly present in Eurasia, whereas ST25 (intermediate virulence) and ST28 (low virulence) strains with a *mrp^−^/epf^+/*−*^/sly^*−*^* profile predominate in North America (Goyette-Desjardins *et al.*, 2014; Lopreto *et al.*, 2005). Interestingly, human cases reported in North America represent less than 0.5 % of described cases worldwide, compared with more than 8 % in European countries, confirming differences in virulence of *S. suis* serotype 2 isolates (Goyette-Desjardins *et al.*, 2014; Auger *et al.*, 2016). Very few data are available concerning serotype 2 strains recovered from diseased pigs in South America (Doto *et al.*, 2016; Martinez* et al*., 2003), where the swine industry is either well-developed (such as in Brazil) or in clear expansion (such as in Argentina).

Herein, we describe new human cases of *S. suis* recovered from diseased patients in Argentina. We have also studied strains isolated from diseased pigs in this country. MLST and pathotype characteristics of strains were determined and representative strains recovered from humans were further analyzed by whole-genome sequencing (WGS).

## Case Report

We present the characteristics of 32* S. suis* strains isolated in Argentina (Table 1). We report and analyze 17 human cases of *S. suis* in Argentina isolated between 1995 and 2016. An additional strain from a previously reported human patient (Lopreto *et al.*, 2005) was also included. All but one of the human strains were isolated from cases of meningitis; the remaining strain was recovered from a patient presenting septic arthritis. Of the 17 cases of meningitis, 7 strains were isolated exclusively from the cerebrospinal fluid (CSF), while the remaining strains were isolated from both CSF and blood. In the latter cases, only one isolate per patient was further characterized. Three isolates were recovered from female patients and 13 patients, from rural areas, recalled having had contact with swine, pork or pork-derived products. Four patients did not recall this information, whereas one patient assured us of having had no such contact.

**Table 1.** *S. suis* strains isolated from humans or pigs in Argentina included in this study

Strain	Source*	Clinical manifestation	Serotype	Host	Date	Gender	Swine/pork contact	Pathotype	ST
285†	CSF, blood	Meningitis	2	H	1995	M	Yes	mrp^+^/epf^+^/sly^+^	1
284	CSF, blood	Meningitis	2	H	1995	M	Yes	mrp^+^/epf^+^/sly^+^	1
263†	CSF, blood	Meningitis	2	H	2003	M	Yes	mrp^+^/epf^+^/sly^+^	1
178†	CSF, blood	Meningitis	2	H	2003	M	Yes	mrp^+^/epf^+^/sly^+^	1
247†	CSF, blood	Meningitis	2	H	2003	M	Yes	mrp^+^/epf^+^/sly^+^	1
2376‡	CSF	Meningitis	2	H	2004	F	Yes	mrp^+^/epf^+^/sly^+^	1
12†	CSF, blood	Meningitis	2	H	2009	M	Yes	mrp^+^/epf^+^/sly^+^	1
83	CSF, blood	Meningitis	2	H	2009	M	Unknown	mrp^+^/epf^+^/sly^+^	1
88	CSF	Meningitis	2	H	2009	M	Yes	mrp^+^/epf^+^/sly^+^	1
245†	CSF	Meningitis	2	H	2012	M	Yes	mrp^+^/epf^+^/sly^+^	1
486†	CSF, blood	Meningitis	2	H	2012	M	Unknown	mrp^+^/epf^+^/sly^+^	1
371†	CSF, blood	Meningitis	2	H	2013	M	Yes	mrp^+^/epf^+^/sly^+^	1
473†	CSF	Meningitis	2	H	2013	F	Unknown	mrp^+^/epf^+^/sly^+^	1
42	CSF, blood	Meningitis	2	H	2014	F	Unknown	mrp^+^/epf^+^/sly^+^	1
130/15	CSF	Meningitis	2	H	2015	M	Yes	mrp^+^/epf^+^/sly^+^	1
695-15	CSF	Meningitis	2	H	2015	M	Yes	mrp^+^/epf^+^/sly^+^	1
136-16	Joint	Arthritis	2	H	2016	M	No	mrp^+^/epf^+^/sly^+^	1
**15**§	CSF	Meningitis	5	H	2014	M	Yes	nd	nd
P156	Spleen	Septicemia	2	S	2000	na	na	mrp^+^/epf^+^/sly^+^	1
P232	Joint	Arthritis	2	S	2001	na	na	mrp^+^/epf^+^/sly^+^	1
050798	Brain	Meningitis	2	S	2001	na	na	mrp^+^/epf^+^/sly^+^	1
P421	Joint	Arthritis	2	S	2002	na	na	mrp^+^/epf^+^/sly^+^	1
130387	Brain	Meningitis	2	S	2002	na	na	mrp^+^/epf^+^/sly^+^	1
P517	Brain	Meningitis	2	S	2003	na	na	mrp^+^/epf^+^/sly^+^	1
477	Heart	Endocarditis	2	S	2003	na	na	mrp^+^/epf^+^/sly^+^	1
P613	Brain	Meningitis	2	S	2005	na	na	mrp^+^/epf^+^/sly^+^	1
P655	Heart	Endocarditis	2	S	2005	na	na	mrp^+^/epf^+^/sly^+^	1
P574	Joint	Arthritis	2	S	2005	na	na	mrp^+^/epf^+^/sly^+^	1
P611	Brain	Meningitis	2	S	2005	na	na	mrp^+^/epf^+^/sly^+^	1
P706†	Lungs	Pneumonia	2	S	2006	na	na	mrp^+^/epf^−^/sly^−^	28
50703-8	Brain	Meningitis	2	S	2010	na	na	mrp^+^/epf^−^/sly^−^	1
BE 21¶	Tonsils	None	2	S	2014	na	na	mrp^+^/epf^−^/sly^−^	28

*When two sources of isolates are mentioned for the same patient, only one strain was characterized; †Strains studied by whole-genome sequencing (see Table 2); ‡(Lopreto *et al*., 2005); §Fatal case; **¶**Clinically healthy pig. ST, sequence type (as evaluated by multilocus sequence typing); CSF, cerebrospinal fluid; nd, not determined; na, not applicable.

We also studied 14 *S. suis* serotype 2 strains recovered from pigs, as pure cultures, from non-related farms: 12 were from systemic disease (meningitis, arthritis or endocarditis). In addition, a strain isolated from the lungs of an animal with pneumonia and another strain isolated from the tonsils of a clinically healthy animal were included.

## Investigations

Identification of *S. suis* by PCR and multiplex PCR was performed as previously described (Okura *et al.*, 2014). Strains positive for serotype 2 were further differentiated from those positive for serotype 1/2 by coagglutination test (Gottschalk *et al.*, 1993). All but one strain from human cases of infection were serotype 2; the remaining strain (the only fatal case) was characterized as serotype 5. No further characterization of this strain was completed.

To evaluate pathotype based on the presence or absence of the traditional virulence factor genes *mrp*,* epf* and *sly*, specific PCRs were performed on serotype 2 strains as previously described (Silva *et al.*, 2006). Interestingly, all serotype 2 strains isolated from humans present the *mrp^+^/epf^+^/sly^+^* profile usually associated with virulent Eurasian serotype 2 strains (Goyette-Desjardins *et al.*, 2014). The *epf^+^* genotype obtained refers to the variant encoding the 110 kDa protein (Smith *et al.*, 1993). All but two strains recovered from pigs also presented this pathotype. The two exceptions were the strain isolated from lungs and that recovered from tonsils of a clinically healthy animal, which both presented the *mrp^+^/epf^−^/sly^−^*profile usually found in North America.

Results from MLST studies, performed as previously described (King *et al.*, 2002), confirm such results. All strains presenting the *mrp^+^/epf^+^/sly^+^* pathotype were ST1, similar to most virulent Eurasian strains (Goyette-Desjardins *et al.*, 2014). The two swine strains presenting the *mrp^+^/epf^−^/sly^−^* profile (one recovered from lungs and the other from tonsils of a clinically healthy animal) were ST28, which is usually associated with low virulence strains in Canada and the USA (Goyette-Desjardins *et al.*, 2014).

**Fig. 1.**
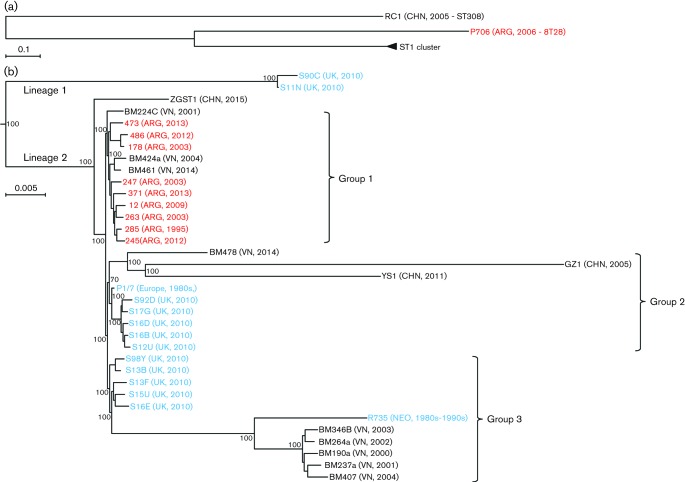
Phylogenetic relationship of ST1 strains. (a) Phylogenetic tree of all strains (including sequence type) used in this study. The ST1 cluster was compressed and is indicated by a triangle. (b) Phylogenetic tree of ST1 strains included in the present study compared with available data from GenBank. The numbers on the branches correspond to the bootstrap values. Strains from different areas are represented by different colors: Asia (black), Europe (blue) and Argentina (red). Geographical origins and year of isolation are included, in parentheses, after the strain name.

Since almost all Argentinean serotype 2 strains from humans and diseased pigs included in this study were ST1, we further compared nine available human ST1 strains and one porcine ST28 strain by WGS. Phylogenetic analyses were performed and compared with the available assembled GenBank genome sequence read data of 26 ST1 strains from the United Kingdom (13 strains), the Netherlands (one strain, reference strain), PR China (three strains), and Vietnam (nine strains) (Table 2). In addition, a Chinese serotype 8 strain was also included as an outsider control. The sequences of strain P1/7 (UK), GZ1 (PR China), BM407 (Vietnam) and R735 (the Netherlands) are completely finished. The methodology used was that previously described (Chen *et al.*, 2013). For each strain, a 500 bp library was constructed and then sequenced using the Hiseq 4000 system (Illumina) to produce 150 bp paired-end reads. The high-throughput read data were mapped to the reference genome of *S. suis* ST1 strain P1/7 (Accession number: NC_012925) using SOAP2 and SNPs detected using SOAPsnp v1.03 (Li *et al.*, 2008). For the assembled genome sequences, SNPs were called using MUMmer v3.23 (Delcher *et al.*, 2003). These SNP patterns were distilled and concatenated using an automatic pipeline as previously described (Chen *et al.*, 2013). Based on this, a phylogenetic tree was reconstructed using the maximum-likelihood method and GTRGAMMA substitution model via raxml software (version 7.2.8) with 1000 bootstrap replications (Stamatakis, 2006). (Fig. 1). The sequencing data were deposited in the GenBank database (Accession number SRP079937).

**Table 2.** List of strains used for comparison of the whole-genome sequencing of *S. suis* from Argentina (see Table 1)

Strain number	Country	ST	Year	Host	Clinical signs
S90C	UK	1	2010	Pig	Diseased
S11N	UK	1	2010	Pig	Diseased
S98Y	UK	1	2010	Pig	Diseased
S13B	UK	1	2010	Pig	Diseased
S16E	UK	1	2010	Pig	Diseased
S15U	UK	1	2010	Pig	Diseased
S13F	UK	1	2010	Pig	Diseased
P1/7	UK	1	1980	Pig	Diseased
S92D	UK	1	2010	Pig	Diseased
S17G	UK	1	2010	Pig	Diseased
S16D	UK	1	2010	Pig	Diseased
S16B	UK	1	2010	Pig	Diseased
S12U	UK	1	2010	Pig	Diseased
R735	Netherlands	1	1963	Pig	Meningitis
YS1	China	1	2011	Pig	Healthy
GZ1	China	1	2005	Human	Meningitis
ZGST1	China	1	2015	Human	Meningitis
RC1*	China	308	2005	Pig	Healthy
BM237a	Vietnam	1	2001	Human	Meningitis
BM407	Vietnam	1	2004	Human	Meningitis
BM190a	Vietnam	1	2000	Human	Meningitis
BM264a	Vietnam	1	2002	Human	Meningitis
BM346B	Vietnam	1	2003	Human	Meningitis
BM478	Vietnam	1	2014	Human	Meningitis
BM224C	Vietnam	1	2001	Human	Meningitis
BM461	Vietnam	1	2014	Human	Meningitis
BM424a	Vietnam	1	2004	Human	Meningitis

*Serotype 8 (outgroup)

Approximately 1.36–1.63 Gb high-quality read data was obtained for each strain and covered 680–858-fold (769±45) of the complete genome of P1/7. A phylogenetic study showed that all of the ST1 strains clustered tightly together when compared with the ST28 strain P706 and ST308 (RC1, PR China) strain (Fig. 1a). Within the ST1 cluster, the nine Argentinean ST1 strains tested clustered together with three other strains from Vietnam, with this cluster being close to another one composed of 11 strains from the UK (Fig. 1b). These two clusters are relatively far away from other European (UK and the Netherlands) and Asian (China and Vietnam) strains.

## Discussion

It has been clearly shown that serotype 2 strains isolated in North America (Canada and the USA) are phenotypically and genotypically highly different from those recovered in Europe and Asia (Fittipaldi *et al.*, 2011). Preliminary results with archetypal strains (Fittipaldi *et al.*, 2011), as well as more recent results with field strains (Auger *et al.*, 2016; Athey *et al.*, 2015), showed that North American *S. suis* serotype 2 strains are, in general, of low virulence. It has been strongly suggested that the relatively low number of human cases in both Canada and the USA (which together represent the second largest swine producing region worldwide after China) are probably due to the lower virulence properties of *S. suis* ST25 and ST28 strains that predominate in these countries.

Very few data are available regarding strains isolated in South America. A few reports on serotype 2 strains recovered from diseased pigs in Brazil have been published (Costa *et al.*, 2005; Doto *et al.*, 2016; Rocha *et al.*, 2012), though no human case has yet been reported. Human cases have been reported in Argentina (Lopreto *et al.*, 2005; Callejo *et al*., 2014; Nagel *et al.*, 2008) and, only once, in Chile (Alarcon *et al.*, 2013), but strains were not further characterized. Although not a traditional pork-producing country, Argentina has significantly increased its swine production in the last few years, having presently more than 4 000 000 pigs (http://www.porcinos.org.ar/). In this study, we analyzed 18 *S. suis* strains isolated from humans, 17 of which were serotype 2. With the addition of another case for which the strain was not available (Nagel *et al.*, 2008) and a serotype 21 strain previously described by us (Callejo *et al*., 2014), this number is considerably higher than in the important swine producing countries of Canada and the USA. Three different factors may explain these differences: (a) the presence of backyard types of swine production; (b) a good surveillance system in rural hospitals and (c) the presence of potentially virulent strains, when compared with those found in North America.

Relatively close contact with pigs/pork products and animals slaughtered at home cannot be ruled out as a possible factor contributing to this atypically high prevalence. More than 75 % of farms in Argentina have fewer than 10 sows (http://www.porcinos.org.ar/), indicating a relatively high number of backyard family types of production with which human *S. suis* infections have been traditionally associated (Huong *et al.*, 2014). Most patients described in this study had contact with swine/pork products and/or live in rural areas. However, it is important to note that typical dishes using raw blood or meat (such as are commonly consumed in some Asian countries) do not exist in the Argentinean culture (Gottschalk *et al.*, 2010). In addition, and since the first human case of *S. suis* has been described more than 10 years ago (Lopreto *et al.*, 2005), a good surveillance system for the characterization of alpha-hemolytic bacteria recovered from cases of meningitis in rural hospitals has been established. Each suspicious isolate is immediately sent to the National Institute of Microbiology for further identification. Consequently, close contact with pigs/pork and a good surveillance system may have contributed to the proper identification of these cases.

However, in the present study, we also demonstrated that serotype 2 strains recovered from either ill patients or systemically diseased pigs in Argentina are mostly ST1 strains with a typical *mrp^+^/epf^+^/sly^+^*genotype that characterizes virulent Eurasian strains. This markedly differs from lower virulence ST25/ST28 North American strains, which have a *mrp*^+/−^/*epf^−^/sly^−^* genotype (Fittipaldi *et al.*, 2011). WGS results indicated that two strains from the UK (S90C and S11N) are clustered together in lineage 1 and significantly diverged from other strains of lineage 2 (Fig. 1b). These two lineages may have evolved from the ancestor of ST1 before separating, with lineage 2 having become dominant. Within this lineage, most strains could be clustered into three main groups with the exception of the Chinese strain ZGST1. Argentinean strains and three strains from Vietnam were clustered into group 1. Strains from the UK, two Chinese strains, other Vietnamese strains, and the old reference strain from the Netherlands were clustered into groups 2 and 3. A reasonable hypothesis is that the ST1 originated in Europe (UK) where a dominant lineage evolved before spreading to other countries maybe via the introduction of animals from genetic companies. In many countries, including China, Vietnam and Argentina, the advanced breeds of pigs have been introduced from European countries like the UK and Denmark. Interestingly, the strains in these three groups of lineage 2 were all isolated in more than one country from different continents. It would appear that these groups diverged before the spreading and were transmitted, in parallel, to different countries.

Interestingly, all strains from diseased pigs in this study were also ST1. Since some genetic breeders from North America have also been incorporated in Argentina, it is possible that North-American-like serotype 2 strains are also present. In fact, one strain isolated from a diseased pig (pneumonia, lungs) and another recovered from tonsils of a clinically healthy pig were typical ST28 strains with a *mrp^+^/epf^−^/sly^−^* genotype profile identical to that found in Canada and the USA (Fittipaldi *et al.*, 2011). In this study, the strain isolated from lungs grouped far from all ST1 strains (Fig. 1a). These strains are most probably of low virulence; it has been previously reported that *S. suis* is not a primary cause of pneumonia and isolates recovered from lungs are often low-virulence (Gottschalk, 2012). It is important to note that these strains are also able to induce serious disease in Canada and the USA. The most important difference between these countries and Argentina, from the disease status point of view, is the absence of the most important swine virus from this South American country: the porcine reproductive and respiratory syndrome virus, which is considered one of the most important predisposing factors for *S. suis* infection (Gottschalk, 2012). In the absence of this virus, only virulent *S. suis* strains are usually able to cause important disease in swine. Finally, transmission of ST1 strains from South America to North America is probably low since pig flow from genetic companies and breeders is usually from Europe and North America to South America.

Finally, we report the first human case of serotype 5 in Argentina (the only fatal case in the present study). Although this is the first report of this serotype in South America, one case of septic arthritis and one case of peritonitis have been previously described in Sweden and Thailand, respectively (Kerdsin *et al.*, 2011; Gustavsson *et al.*, 2014). In addition, one human case of arthroplasty infection with streptococcal toxic shock-like syndrome, was caused by a non-encapsulated strain belonging to this serotype in the USA (Gomez *et al.*, 2014).

In conclusion, and in addition to the probably close contact with pigs/pork products and a good surveillance system, the presence of potentially virulent Eurasian-like serotype 2 strains in Argentina may be an important risk factor contributing to the higher number of human cases observed. In fact, and with this report, Argentina is now among the Western countries with the highest number of reported human cases after the Netherlands, France and the UK, with a similar number of cases to Poland (Bojarska *et al.*, 2016). However, the three former European countries began the identification of *S. suis* in humans more than 15 years before Argentina, so the total number of human cases in this South American country may have been underestimated. Further studies in other South American countries, such as Brazil, where swine production is very important, should be performed.
